# Comparison of Perioperative Outcomes Between Conventional Laparoscopic Approach and Robot-Assisted Approach in Revision and Conversion Surgery: Experience at the National Medical Center “20 de Noviembre”

**DOI:** 10.7759/cureus.105383

**Published:** 2026-03-17

**Authors:** Jesús Montoya Ramírez, Juan Sánchez Lora, Hugo Fernando Narvaez Gonzalez, Carlos Felipe Acuña Cota, José Francisco Rodríguez Salinas, Dania Ramírez González, Rommel Ramírez López, Jhon Mauricio Coronel Ruilova

**Affiliations:** 1 Bariatric Surgery, Centro Medico Nacional 20 de Noviembre, Mexico City, MEX; 2 Gastrointestinal Endoscopy, Centro Medico Nacional 20 de Noviembre, Mexico City, MEX; 3 Surgery, Hospital de Especialidades de la Ciudad de México Dr. Belisario Domínguez, Mexico City, MEX; 4 Surgery, Hospital General Monte Sinaí, Guayaquil, ECU

**Keywords:** bariatric surgery, recurrent weight gain, revision and conversion surgery, robot-assisted bariatric surgery, roux-en-y gastric bypass

## Abstract

Introduction

Revision and conversion surgery (RCS) is technically demanding and associated with higher perioperative risk compared with primary bariatric procedures. The increasing adoption of robotic technology has expanded surgical options for complex revisional cases; however, comparative data from public healthcare institutions remain limited. This study aimed to compare perioperative outcomes between conventional laparoscopic and robot-assisted laparoscopic approaches (RALA) in patients undergoing RCS at a tertiary referral public center.

Methods

An observational, analytical, retrospective comparative study was conducted at the National Medical Center “20 de Noviembre,” Instituto de Seguridad y Servicios Sociales de los Trabajadores del Estado (ISSSTE), Mexico City, including patients who underwent bariatric revisional surgery between January 2021 and December 2024 using either a conventional laparoscopic or RALA. Perioperative outcomes were compared between groups. Continuous variables were analyzed using Student’s t-test or the Mann-Whitney U test, and categorical variables using the chi-square test, with statistical significance set at p < 0.05.

Results

A total of 90 patients underwent bariatric revisional surgery. The RALA was associated with significantly longer operative time than conventional laparoscopy (193.1 ± 25 vs 145.6 ± 18 minutes, p < 0.0001). Overall complication rates were low (3.3%), with no significant differences between groups, and no mortality was observed. Conversion rates were comparable between approaches.

Conclusion

In a high-volume public referral center, both conventional laparoscopic and robot-assisted RCS demonstrated favorable perioperative outcomes. Although operative time was significantly longer with the RALA, complication rates were comparable, with no mortality reported. These findings suggest that robot-assisted RCS may be a feasible approach in specialized centers; however, further prospective studies with larger cohorts and longer follow-up are needed to better define the comparative role of robotic and conventional laparoscopic approaches.

## Introduction

Metabolic bariatric surgery (MBS) has proven to be the most effective therapeutic modality for achieving sustained weight loss and improving multiple obesity-related health conditions, such as type 2 diabetes (T2D), hypertension, and various types of dyslipidemia [1-3]. However, a percentage of patients undergoing primary bariatric surgery may experience insufficient weight loss during the weight loss process, resulting in recurrent weight regain or complications requiring surgical re-exploration. Such procedures are referred to as revision and conversion surgery (RCS) [4-6].

RCS refers to any surgical intervention performed after an initial MBS to correct technical failures, address complications, and improve metabolic or weight-loss outcomes [6]. The primary indications for such revisions include recurrent weight regain, insufficient weight loss, and complications such as fistulas, strictures, severe gastroesophageal reflux disease (GERD), and internal hernias, among others [4,5].

In Mexico, a sustained increase in these re-interventions has been documented. Hernández et al. identified refractory GERD and recurrent weight gain as the main reasons for revision surgery, directly impacting hospital readmission rates and postoperative morbidity [7].

The laparoscopic approach has been the standard in bariatric surgery for several decades due to its numerous benefits over laparotomy [8]. In recent years, the robot-assisted laparoscopic approach (RALA) has gained popularity, particularly for complex procedures such as RCS, owing to its technical advantages, including three-dimensional visualization, enhanced precision, and improved surgeon ergonomics [9,10].

Recent studies have reported that the RALA may be associated with lower rates of conversion to open surgery, reduced intraoperative blood loss, and greater ease in performing complex anastomoses [11,12]. However, its implementation also presents challenges, including high costs, longer operating times in some scenarios, and the need for trained personnel and well-structured institutional systems [9,13].

Given the growing need for revision procedures and continuous advances in surgical technology, it is essential to systematically evaluate the outcomes of both approaches at a national referral center. The aim of this study was to compare perioperative outcomes between conventional laparoscopic and RALA in patients undergoing revision and conversion bariatric surgery at a tertiary referral public center. Specifically, operative time, conversion rates, postoperative complications, and length of hospital stay were evaluated as key perioperative outcomes. We hypothesized that robot-assisted surgery would demonstrate perioperative safety outcomes comparable to those of conventional laparoscopy, although operative time may differ between approaches. By examining these outcomes within a single high-volume institutional setting, this study seeks to contribute comparative data on the feasibility and safety of robotic technology in RCS.

## Materials and methods

An observational, analytical, retrospective, and comparative study was conducted by reviewing clinical records and institutional databases to evaluate perioperative outcomes in patients undergoing RCS. Patients were identified through the institutional bariatric surgery registry and cross-referenced with operative reports in the hospital's electronic medical record system. Patients who underwent RCS using either a conventional laparoscopic approach or a RALA at the National Medical Center “20 de Noviembre,” ISSSTE, a tertiary referral center in Mexico City, between January 1, 2021, and December 31, 2024, were included. The selection of surgical approach was based on institutional robotic platform availability at the time of surgery, surgeon preference, and case complexity as assessed during the preoperative multidisciplinary evaluation. Robot-assisted procedures were performed using the Da Vinci Surgical System (Intuitive Surgical, Sunnyvale, CA, USA).

The study population consisted of ISSSTE beneficiaries who had undergone primary metabolic and bariatric surgery and subsequently required RCS during the study period. Eligible patients had complete clinical records, including operative reports and postoperative follow-up for up to 30 days. Patients were excluded if duplicate records were identified or if there was insufficient documentation regarding intraoperative conversion to open surgery. A convenience sampling strategy was used. Data were collected using standardized institutional tools to ensure systematic and reliable acquisition. Variables analyzed included demographic characteristics, operative time, postoperative complications within 30 days, length of hospital stay, and conversion to open surgery. Postoperative complications occurring within 30 days were recorded based on clinical documentation in the medical record. All patients were managed according to standardized institutional perioperative protocols for bariatric surgery, including thromboprophylaxis, early mobilization, and progressive postoperative dietary advancement.

Statistical analyses were performed using IBM SPSS Statistics version 26.0 (IBM Corp., Armonk, NY, USA), with data management conducted in Microsoft Excel for Mac version 16.99 (Microsoft Corp., Redmond, WA, USA). Continuous variables were summarized using measures of central tendency and dispersion and compared between groups using Student’s t-test or the Mann-Whitney U test, as appropriate based on data distribution. Categorical variables were expressed as frequencies and percentages and compared using the chi-square test. Statistical significance was defined as a two-sided p-value < 0.05.

Ethical considerations

This study was conducted in accordance with the ethical principles outlined in the Declaration of Helsinki. Given the retrospective nature of the study and the use of anonymized data obtained from institutional medical records, the Institutional Review Board of the National Medical Center “20 de Noviembre” ISSSTE waived the requirement for informed consent as part of the ethical approval process. The study protocol was reviewed and approved by the Institutional Review Board and Ethics Committee of the National Medical Center “20 de Noviembre” ISSSTE, under approval number 123.2025, assigned by the Research Department following the institutional review process. Patient confidentiality was strictly maintained throughout data collection and analysis.

## Results

A total of 90 patients were included in the analysis. Baseline demographic and clinical characteristics of the study population are summarized in Table 1. The mean age was 44.6 ± 13 years, and most patients were female, a total of 63 patients (70%). The mean body weight was 115.3 ± 15 kg, and the mean body mass index was 43.4 ± 6 kg/m². All patients were classified as American Society of Anesthesiologists physical status III [14].

**Table 1:** Baseline characteristics of the study population Data are presented as mean ± standard deviation or n (%), as appropriate.

Variable	Value
Number of patients	n= 90
Sex	Female: 63 (70.0%)
	Male: 27 (30.0%)
Age (years)	44.6 ± 13
Body mass index (BMI), kg/m²	43.4 ± 6
Weight (kg)	115.3 ± 15

Regarding the surgical approach, 62 patients (68.9%) underwent robot-assisted RCS, while 28 patients (31.1%) were scheduled for conventional laparoscopic RCS. Among the robotic cases, conversion from robotic to laparoscopic surgery was required in five patients (8.1% of the RALA group, representing 5.6% of the overall cohort), whereas no conversions to open surgery were recorded in either surgical group. The most frequently performed revisional procedure was conversion from sleeve gastrectomy to Roux-en-Y gastric bypass (RYGB) due to weight regain, performed in 79 patients (87.8%). Conversion from gastric banding to RYGB was performed in eight patients (8.8%). Structural abnormalities accounted for 11 cases (12.2%) as an indication for revision surgery. The mean time interval between the primary bariatric procedure and RCS was 11.8 ± 3.25 months.

Perioperative outcomes and surgical characteristics are detailed in Table 2. Operative time differed significantly between surgical approaches. The mean operative time for robot-assisted RCS was 193.1 ± 25 minutes, compared with 145.6 ± 18 minutes for conventional laparoscopic RCS. Using an independent-samples t-test with Welch’s correction for unequal variances, this difference was statistically significant (t = 9.99, p < 0.0001). A chi-square test was performed to compare conversion rates between the robot-assisted and conventional laparoscopic groups. Although conversions occurred only in the robot-assisted group (5 cases), the difference was not statistically significant (χ² = 2.39, p = 0.12).

**Table 2:** Perioperative outcomes and surgical characteristics. Data are presented as mean ± standard deviation or n (%), as appropriate.

Variable	Value
Operative time (minutes)	Robot-assisted: 193.1 ± 25
	Conventional laparoscopy: 145.6 ± 18
Conversion from robotic to laparoscopic surgery	5 (8.1%)
Length of hospital stay (days)	2.1 ± 1
Time from primary surgery to bariatric revision (months)	11.8 ± 3
Indication for revision and conversion surgery	Weight regain: 79 (87.8%)
	Structural abnormality: 11 (12.2%)
Mortality	0 (0.0%)
Complications	3 (3.3%)

The mean postoperative hospital stay was 2.1 ± 1 day. Postoperative complications occurred in three patients (3.3%), all related to intraoperative bleeding exceeding 400 ml. All complications occurred in the conventional laparoscopic group and were managed intraoperatively without the need for conversion to open surgery. No patients required reoperation during the 30-day follow-up period. Additionally, no cases of gastrointestinal perforation, vascular injury, or postoperative mortality were recorded.

## Discussion

Obesity is a chronic, multifactorial, and progressive disease that affects more than 70% of the adult population in Mexico, with prevalence continuing to increase [7]. It is associated with a significantly higher risk of morbidity and mortality, as well as the development of multiple comorbidities, including T2D, systemic hypertension, dyslipidemia, and obstructive sleep apnea [1,2]. Conventional medical management has demonstrated limited long-term efficacy, which is why bariatric surgery has been established as the most effective therapeutic strategy for sustained weight loss and metabolic improvement [1,3].

The evolution of bariatric surgery dates back to the 1950s, with the introduction of highly malabsorptive procedures such as jejunoileal bypass, which were later abandoned due to severe metabolic and nutritional complications [15]. Over time, restrictive techniques such as vertical banded gastroplasty and adjustable gastric banding were developed, followed by combined procedures such as RYGB and sleeve gastrectomy, which demonstrated superior risk-benefit profiles and more durable outcomes [16,17]. Parallel advances in surgical technology, anesthetic safety, patient selection, and postoperative follow-up have contributed to progressive improvements in bariatric surgery outcomes worldwide [3,15].

Currently, RYGB and sleeve gastrectomy are the most frequently performed bariatric procedures globally [16,17]. However, the increasing volume of primary bariatric surgeries has inevitably led to a growing demand for RCS. Approximately 10%-28% of patients undergoing bariatric surgery will require RCS during long-term follow-up due to weight regain, insufficient weight loss, or procedure-related complications [13,18].

The primary indications for RCS include new weight gain following an initial successful weight loss, inadequate weight loss after the primary procedure, anatomical complications such as strictures, fistulas, or severe GERD, and the persistence or recurrence of metabolic comorbidities [4,7,17,19]. In Mexico, Hernández et al. reported that weight regain and refractory GERD are among the most common reasons for surgical reintervention, with a direct impact on postoperative morbidity and hospital readmission rates [7]. In our study, the main indication was recurrent weight regain, observed in 79 patients (87.8%) who underwent sleeve gastrectomy to RYGB, consistent with the literature. The relatively short interval observed between the primary bariatric procedure and revisional surgery in our cohort may reflect the underlying indications for conversion. In many cases, revisional procedures were performed due to early postoperative complications or persistent symptoms, particularly severe GERD following sleeve gastrectomy. These clinical scenarios may require earlier surgical intervention compared with revisions performed for long-term weight regain.

RCS is inherently more complex than primary bariatric procedures due to anatomical alterations from previous interventions, the presence of adhesions, and the need to identify structures that have already been modified by prior surgery [5,6]. Consequently, perioperative complication rates are higher than those observed in primary surgery. Multicenter studies have reported major complication rates ranging from 5% to 20%, intraoperative hemorrhage in 1-5% of cases, average hospital stays of 2-5 days, mortality rates below 1% in high-volume centers, and increased rates of reintervention or reoperation [11-13,19]. In our cohort, the complication rate was low, with bleeding-related events occurring in 3.3% of patients, which is within the range reported in the literature; however, hospital stay was shorter in our series.

Reported outcomes of RCS vary with the revision technique used. A systematic review and network meta-analysis by Chierici et al. demonstrated that, although duodenal switch-based revisions may achieve superior weight loss, RYGB has lower postoperative morbidity and a more favorable safety profile when performed as a revisional procedure [19]. In our cohort, the most commonly performed RCS was RYGB. No duodenal switch procedures were performed at our center during the study period. Although duodenal switch-based revisions may achieve greater weight-loss outcomes in selected patient populations, this approach is also associated with higher rates of postoperative morbidity and nutritional complications. Therefore, RYGB remains a widely adopted revisional strategy due to its favorable balance between efficacy and safety.

The advent of robotic surgical technology has expanded the therapeutic options for complex bariatric procedures. The Da Vinci robotic system provides high-definition three-dimensional visualization, articulated instruments with seven degrees of freedom, and enhanced precision for dissection and suturing, features that are particularly advantageous in technically demanding surgeries such as bariatric revision [9,10].

Comparative studies by Bertoni et al. and Clapp et al. have evaluated robotic-assisted versus conventional laparoscopic RCS, reporting lower conversion rates to open surgery and reduced intraoperative blood loss with the robotic approach, albeit at the expense of longer operative times and increased costs [20,21]. Overall, complication rates and lengths of hospital stay appear comparable between both approaches when performed in experienced centers [11,21]. In our cohort, operative time was also longer in the robot-assisted group, while complication rates and hospital stay were comparable between approaches. Because quantitative intraoperative blood loss was not consistently documented, this variable could not be directly compared in our study.

Long-term follow-up studies after sleeve gastrectomy have shown that a subset of patients may require conversion to RYGB due to weight-related failure or reflux-related symptoms [22]. Additionally, recent experiences from high-volume bariatric centers have described laparoscopic revisional procedures after one anastomosis gastric bypass, supporting the feasibility and safety of revision surgery in specialized units with structured protocols [23].

In the present series, both surgical approaches demonstrated favorable perioperative outcomes, with low complication rates and no mortality. Although the RALA was associated with significantly longer operative times, this did not translate into increased morbidity. The absence of conversions to open surgery and the limited need for conversion from robotic to conventional laparoscopy underscore the importance of surgical expertise, appropriate patient selection, and institutional experience over the exclusive use of advanced technology [8,9].

These findings suggest the feasibility and safety of robot-assisted RCS as a viable alternative for selected patients when performed in high-volume centers with experienced multidisciplinary teams. Further prospective studies are needed to better define metabolic outcomes, cost-effectiveness, and optimal patient selection criteria for each surgical approach. In this context, validating perioperative risk prediction tools specifically designed for RCS may further strengthen clinical decision-making and optimize outcomes. [24-28].

Strengths and limitations

This study has strengths that should be highlighted. First, it provides institutional data from a high-volume tertiary referral center within the Mexican public healthcare system, where access to robotic technology remains limited. This provides valuable real-world evidence from a setting underrepresented in the international literature on RCS. Second, the comparison between conventional laparoscopic and RALA was conducted within the same institutional framework, minimizing variability in perioperative protocols, patient selection, and multidisciplinary care. Third, all procedures were performed by an experienced bariatric surgeon, which may enhance procedural consistency and internal validity; however, this single-surgeon experience may limit the generalizability of the findings to other surgical settings.

Additionally, this study addresses a clinically relevant and increasingly frequent scenario, given the growing demand for RCS worldwide. The analysis of perioperative outcomes contributes to the ongoing discussion regarding the feasibility, safety, and practical implications of adopting robotic technology for complex revisional procedures, particularly in resource-constrained public health systems.

However, several limitations must be acknowledged. The retrospective design inherently limits causal inference and is subject to selection bias. The allocation to robotic or conventional laparoscopy was not randomized and depended on institutional availability, surgeon preference, and case complexity, which may have influenced comparability between groups. Although both groups were managed within the same institutional framework, residual confounding cannot be excluded. Future prospective studies with larger cohorts may enable more robust statistical adjustments, such as propensity score matching or multivariable modeling, to better control for potential selection bias.

Additionally, the relatively small sample size, particularly in the conventional laparoscopic group, may limit the statistical power to detect differences in low-frequency outcomes such as perioperative complications. Because this study was designed as a retrospective analysis of all eligible cases during the study period, a formal a priori sample size calculation was not performed. Additionally, the imbalance in group sizes between the robot-assisted and conventional laparoscopic cohorts may influence the statistical power of between-group comparisons and should be considered when interpreting the reported outcomes.

The use of convenience sampling further limits the external validity of the findings, as the study reflects the experience of a single tertiary referral center within the Mexican public healthcare system. Consequently, the generalizability of these results to other institutions, particularly those with different surgical volumes or access to robotic platforms, may be limited.

Furthermore, due to the retrospective nature of the study, quantitative intraoperative blood loss was not consistently documented in operative reports. For this reason, only clinically significant bleeding events were analyzed, which may limit the ability to perform a detailed comparison of intraoperative blood loss between surgical approaches. In addition, postoperative complications were recorded based on clinical documentation in the medical record; however, a standardized complication classification system such as the Clavien-Dindo grading system was not applied, which may limit comparability with other studies in the surgical literature.

Finally, the follow-up period was limited to 30 days and therefore focused primarily on short-term perioperative outcomes. Long-term outcomes, including weight-loss efficacy, metabolic improvement, quality of life, and cost-effectiveness, were not evaluated, although these parameters are essential for a comprehensive assessment of revision and conversion surgery.

Despite these limitations, the results provide meaningful insight into the real-world performance of robotic-assisted RCS and support its role as a safe and feasible alternative when performed in specialized centers with appropriate expertise and infrastructure.

Future research should prioritize evaluating long-term outcomes following RCS. In particular, prospective studies assessing sustained weight-loss efficacy, metabolic improvement, patient-reported quality of life, and cost-effectiveness are essential to better define the comparative value of robotic and conventional laparoscopic approaches in metabolic bariatric revisional procedures.

## Conclusions

In this single-center experience at a tertiary public referral hospital, both conventional laparoscopic and robot-assisted revision and conversion bariatric surgery demonstrated low complication rates and no mortality within the 30-day follow-up period. Although the RALA was associated with longer operative times, perioperative outcomes were comparable between techniques. These findings should be interpreted within the limitations of a retrospective, non-randomized study and should be considered hypothesis-generating. Further prospective studies with larger cohorts and longer follow-up are needed to better define the comparative role of robotic and conventional laparoscopic approaches in revisional bariatric surgery, including long-term outcomes and cost-effectiveness.

## References

[1] Buchwald H, Avidor Y, Braunwald E, Jensen MD, Pories W, Fahrbach K, Schoelles K (2004). Bariatric surgery: A systematic review and meta-analysis. JAMA.

[2] Salminen P, Grönroos S, Helmiö M (2022). Effect of laparoscopic sleeve gastrectomy vs Roux-en-Y gastric bypass on weight loss, comorbidities, and reflux at 10 years in adult patients with obesity: The SLEEVEPASS randomized clinical trial. JAMA Surg.

[3] Brethauer SA, Kim J, el Chaar M (2015). Standardized outcomes reporting in metabolic and bariatric surgery. Surg Obes Relat Dis.

[4] Ponce CJ, Guerron AD, Sudan R (2021). Robot-assisted revisional bariatric surgery. Dig Med Res.

[5] Jones KB Jr (2001). Revisional bariatric surgery--safe and effective. Obes Surg.

[6] Mirkin K, Alli VV, Rogers AM (2021). Revisional bariatric surgery. Surg Clin North Am.

[7] Hernández LA, Guilbert L, Sepúlveda EM, Rodríguez F, Peñuñuri F, García VH, Zerrweck C (2023). Causes of revisional surgery, reoperations, and readmissions after bariatric surgery. Rev Gastroenterol Mex (Engl Ed).

[8] Hutter MM, Schirmer BD, Jones DB, Ko CY, Cohen ME, Merkow RP, Nguyen NT (2011). First report from the American College of Surgeons Bariatric Surgery Center Network: Laparoscopic sleeve gastrectomy has morbidity and effectiveness positioned between the band and the bypass. Ann Surg.

[9] Wilson EB (2009). The evolution of robotic general surgery. Scand J Surg.

[10] Switzer NJ, Karmali S, Gill RS, Sherman V (2016). Revisional bariatric surgery. Surg Clin North Am.

[11] Iranmanesh P, Bajwa KS, Felinski MM, Shah SK, Wilson EB (2020). Robotic primary and revisional bariatric surgery. Surg Clin North Am.

[12] Nasser H, Munie S, Kindel TL, Gould JC, Higgins RM (2020). Comparative analysis of robotic versus laparoscopic revisional bariatric surgery: Perioperative outcomes from the MBSAQIP database. Surg Obes Relat Dis.

[13] Dardamanis D, Navez J, Coubeau L, Navez B (2018). A retrospective comparative study of primary versus revisional Roux-en-Y gastric bypass: Long-term results. Obes Surg.

[14] ASA Committee of Oversight: Economics (2026). American Society of Anesthesiologists: Statement on ASA physical status classification system. Anesthesiol Open.

[15] Deitel M, Greenstein RJ (2003). Recommendations for reporting weight loss. Obes Surg.

[16] Angrisani L, Santonicola A, Iovino P (2018). IFSO worldwide survey 2016: Primary, endoluminal, and revisional procedures. Obes Surg.

[17] Felsenreich DM, Artemiou E, Steinlechner K (2021). Fifteen years after sleeve gastrectomy: weight loss, remission of associated medical problems, quality of life, and conversions to Roux-en-Y gastric bypass-long-term follow-up in a multicenter study. Obes Surg.

[18] English WJ, DeMaria EJ, Hutter MM, Kothari SN, Mattar SG, Brethauer SA, Morton JM (2020). American Society for Metabolic and Bariatric Surgery 2018 estimate of metabolic and bariatric procedures performed in the United States. Surg Obes Relat Dis.

[19] Chierici A, Chevalier N, Iannelli A (2022). Postoperative morbidity and weight loss after revisional bariatric surgery for primary failed restrictive procedure: A systematic review and network meta-analysis. Int J Surg.

[20] Bertoni MV, Tabbara M, Genser L (2022). Robotic-assisted versus laparoscopic revisional bariatric surgery: A systematic review and meta-analysis on perioperative outcomes. Obes Surg.

[21] Clapp B, Liggett E, Jones R, Lodeiro C, Dodoo C, Tyroch A (2019). Comparison of robotic revisional weight loss surgery and laparoscopic revisional weight loss surgery using the MBSAQIP database. Surg Obes Relat Dis.

[22] Liagre A, Martini F, Van Haverbeke O, Boudry H, Petrucciani N (2025). Ten-year outcomes of sleeve gastrectomy in a single-center series of 156 patients: Weight loss and need of revisional surgery. Am J Surg.

[23] Conte LE, Sensi B, Griguolo G (2025). Laparoscopic revision after one anastomosis gastric bypass (OAGB): A 4-years experience in a single high-volume bariatric surgery center in northern Italy. Updates Surg.

[24] AbuHasan Q, Chesnova B, Sanduka D, Saeidi S, Chang W, Yuce TK, Stefanidis D (2025). Accuracy of the metabolic and bariatric surgery accreditation quality improvement program risk/benefit calculator in predicting outcomes of revisional bariatric surgery. Surg Obes Relat Dis.

[25] Sánchez-Pernaute A, López-Antoñanzas L, Torres AJ, Dziakova J, Rubio MA, Pérez-Aguirre E (2023). Avoiding complications during revisional bariatric surgery with indocyanine green fluorescence imaging. Obes Surg.

[26] Buchs NC, Pugin F, Azagury DE, Huber O, Chassot G, Morel P (2014). Robotic revisional bariatric surgery: A comparative study with laparoscopic and open surgery. Int J Med Robot.

[27] Khalaj A, Barzin M, Ebadinejad A, Mahdavi M, Ebrahimi N, Valizadeh M, Hosseinpanah F (2023). Revisional bariatric surgery due to complications: Indications and outcomes. Obes Surg.

[28] Karanam VP, Panchal AM, Sepuri SK, Chalamarla LK, Ravula PK (2025). Atypical biliary fistula after revisional bariatric surgery: A case report. J Metab Bariatr Surg.

